# Supplementary material for: From sequence to enzyme mechanism using multi-label machine learning
Source: BMC Bioinformatics. 2014 May 19;15:150. doi: 10.1186/1471-2105-15-150 (PMC4229970; doi:10.1186/1471-2105-15-150)
Supplement: Additional file 2 — Java code of ml2db. Additional file ml2db_code.tar.gz contains the Java source code to run the multi-label machine learning experiments and save the results to database. The code’s Javadoc is included. [file 1471-2105-15-150-S2.zip › additional file 2/ml2db/ml2db/doc/mulan/data/class-use/LabelPairsDependenceIdentifier.html]

Uses of Interface mulan.data.LabelPairsDependenceIdentifier


JavaScript is disabled on your browser.


- Overview
- Package
- Class
- Use
- Tree
- Deprecated
- Index
- Help

- Prev
- Next

- Frames
- No Frames

- All Classes

## Uses of Interface mulan.data.LabelPairsDependenceIdentifier

- Packages that use LabelPairsDependenceIdentifier

  | Package | Description |
  | --- | --- |
  |  |  |
  | --- | --- |
  | mulan.classifier.meta |  |
  | mulan.data |  |
- - ### Uses of LabelPairsDependenceIdentifier in mulan.classifier.meta

    Methods in mulan.classifier.meta with parameters of type LabelPairsDependenceIdentifier

    | Modifier and Type | Method and Description |
    | --- | --- |
    |  |  |
    | --- | --- |
    | `void` | EnsembleOfSubsetLearners.`setDependenceIdentifier(LabelPairsDependenceIdentifier dependenceIdentifier)` |

    Constructors in mulan.classifier.meta with parameters of type LabelPairsDependenceIdentifier

    Constructor and Description

    `EnsembleOfSubsetLearners(MultiLabelLearner aMultiLabelLearner,
    weka.classifiers.Classifier aSingleLabelLearner,
    LabelPairsDependenceIdentifier aDependenceIdentifier,
    int models)`

    Initialize EnsembleOfSubset with multilabel and single label learners, a
    method for labels dependence identification and number of models to
    constitute the ensemble.
  - ### Uses of LabelPairsDependenceIdentifier in mulan.data

    Classes in mulan.data that implement LabelPairsDependenceIdentifier

    | Modifier and Type | Class and Description |
    | --- | --- |
    |  |  |
    | --- | --- |
    | `class` | `ConditionalDependenceIdentifier` A class for identification of conditional dependence between each pair of labels. |
    | `class` | `UnconditionalChiSquareIdentifier` A class for identification of unconditional dependence between each pair of labels using Chi Square Test For Independence. |

    Constructors in mulan.data with parameters of type LabelPairsDependenceIdentifier

    Constructor and Description

    `GreedyLabelClustering(MultiLabelLearner aMultiLabelLearner,
    weka.classifiers.Classifier aSingleLabelLearner,
    LabelPairsDependenceIdentifier dependenceIdentifier)`

    Initialize the GreedyLabelClustering with multilabel and single label learners and a method
    for labels dependence identification.

- Overview
- Package
- Class
- Use
- Tree
- Deprecated
- Index
- Help

- Prev
- Next

- Frames
- No Frames

- All Classes
